# A LacI-Family Regulator Activates Maltodextrin Metabolism of *Enterococcus faecium*

**DOI:** 10.1371/journal.pone.0072285

**Published:** 2013-08-07

**Authors:** Xinglin Zhang, Malbert Rogers, Damien Bierschenk, Marc J. M. Bonten, Rob J. L. Willems, Willem van Schaik

**Affiliations:** Department of Medical Microbiology, University Medical Center Utrecht, Utrecht, The Netherlands; University of Padova, Medical School, Italy

## Abstract

*Enterococcus faecium* is a gut commensal of humans and animals. In the intestinal tract, *E. faecium* will have access to a wide variety of carbohydrates, including maltodextrins and maltose, which are the sugars that result from the enzymatic digestion of starch by host-derived and microbial amylases. In this study, we identified the genetic determinants for maltodextrin utilization of *E. faecium* E1162. We generated a deletion mutant of the *mdxABCD-pulA* gene cluster that is homologous to maltodextrin uptake genes in other Gram-positive bacteria, and a deletion mutant of the *mdxR* gene, which is predicted to encode a LacI family regulator of *mdxABCD-pulA*. Both mutations impaired growth on maltodextrins but had no effect on the growth on maltose and glucose. Comparative transcriptome analysis showed that eight genes (including *mdxABCD-pulA*) were expressed at significantly lower levels in the isogenic Δ*mdxR* mutant strain compared to the parental strain when grown on maltose. Quantitative real-time RT-PCR confirmed the results of transcriptome analysis and showed that the transcription of a putative maltose utilization gene cluster is induced in a semi-defined medium supplemented with maltose but is not regulated by MdxR. Understanding the maltodextrin metabolism of *E. faecium* could yield novel insights into the underlying mechanisms that contribute to the gut commensal lifestyle of *E. faecium*.

## Introduction

Enterococci are facultative anaerobic Gram-positive bacteria commonly found in the gastrointestinal tracts of humans and animals [1]. In the last twenty years, *E. faecium* has emerged as a clinical pathogen of major importance. This development has been linked to its ability to efficiently acquire antibiotic resistance genes and genetic elements that may contribute to virulence [2,3].

The ability of both commensal and clinical *E. faecium* strains to effectively colonize the intestinal tract determines the ecological success of this species. Therefore, understanding the mechanisms of successful host colonization is important for the development of novel strategies to prevent or treat infections with these opportunistic pathogens. The metabolism of carbohydrates in the complicated food webs of the mammalian intestinal tract is crucially important for gut colonization of commensals and opportunistic pathogens [4–8]. Carbohydrate utilization of *E. faecium* remains poorly understood despite its potential importance in colonization and adaptation to healthy individuals [9] and hospitalized patients [10].

One of the main energy and carbon sources for bacteria in the intestine originates from complex polysaccharides, such as starch [4]. Starch is a plant storage glycan that consists of glucose monomers joined via α-1,4 glycosidic linkages with additional branches introduced by α-1,6 linked glucose moieties. In the human intestinal tract, starch is digested by host-derived and microbial amylases. Its breakdown products (mainly maltose and maltodextrins) can be absorbed by the host small intestine [11], but can also reach the colon [12,13] where they can be metabolized by bacteria from several genera [14,15]. The metabolism of maltodextrin has been investigated in *Escherichia coli* [16,17] and in several Gram-positive bacteria, including *Bacillus subtilis* [18,19], *Listeria monocytogenes* [20] and *Streptococcus pyogenes* [21,22]. The maltose/maltodextrin regulon in *E. coli* consists of ten genes encoding four glycoside hydrolases, a maltodextrin phosphorylase, a maltodextrin glucosidase, a periplasmic α-amylase, together with an ATP-binding cassette (ABC) transporter [16,17]. In *B. subtilis*, maltose and maltodextrin are separately transported by a maltose-specific phosphotransferase system and a maltodextrin-specific ABC transporter, respectively [18], while in *L. monocytogenes* both maltose and maltodextrin are taken up by the same ABC transporter [20]. In this study, we identified the determinants of maltodextrin uptake and metabolism in *E. faecium*.

## Materials and Methods

### Bacterial strains, plasmids and growth conditions

*E. faecium* strains, *E. coli* strains and plasmids used or generated in this study are listed in Table 1. The *E. faecium* strain E1162 (with sequence type 17) was used throughout this study. This strain was isolated from a bloodstream infection in France in 1996 and its genome has previously been sequenced [23]. Unless otherwise mentioned, *E. faecium* was grown in brain heart infusion broth (BHI; Oxoid) at 37° C. The *E. coli* strains DH5α (Invitrogen) and EC1000 [24] were grown in Luria-Bertani medium. Where necessary, antibiotics were used at the following concentrations: gentamicin at 300 µg ml^−1^ for *E. faecium* and 25 µg ml^−1^ for *E. coli*, spectinomycin at 300 µg ml^−1^ for *E. faecium* and 100 µg ml^−1^ for *E. coli*. All antibiotics were obtained from Sigma-Aldrich (Saint Louis, MO). Growth of cultures was determined by measuring the optical density at 660 nm (OD_660_).

**Table 1:** Strains and plasmids used in this study.

**Strain or plasmid**	**Relevant characteristic(s)**	**Source or reference**
*E. faecium*		
E1162	Clinical isolate (bloodstream infection), isolated in France, 1996	[23]
Δ*mdxR*	Markerless deletion mutant of *mdxR* of E1162	This study
Δ*mdxABCD-pulA*	Markerless deletion mutant of the *mdxABCD*-*pulA* gene cluster of E1162	This study
Δ*mdxR+mdxR*	Complementation strain of Δ*mdxR*; Δ*mdxR* harboring pEF25-*mdxR*	This study
*E. coli* strains		
DH5*α*	*E. coli* host strain for routine cloning	Invitrogen
EC1000	MC1000 *glgB*::*repA*; host strain for pWS3 derived vectors	[24]
Plasmids		
pWS3	Gram-positive thermosensitive origin of replication; Spc^r^	[9]
pDEL1a	pWS3 carrying the 5′ and 3′ flanking regions of *mdxR* for mutant construction	This study
pDEL2a	pWS3 carrying the 5′ and 3′ flanking regions of *mdxABCD-pulA* gene cluster for mutant construction	This study
pDEL1b	pDEL1a with a Gen^r^ cassette which was flanked by *lox66*- and *lox71*-sites cloned between the 5′ and 3′ flanking regions	This study
pDEL2b	pDEL2a with a Gen^r^ cassette which was flanked by *lox66*- and *lox71*-sites cloned between the 5′ and 3′ flanking regions	This study
pWS3-Cre	pWS3 derivative expressing Cre in *E. faecium*	[26]
pEF25	Shuttle plasmid pAT18 with spectinomycin resistance cassette cloned in the EcoRI site; Spc^r^ Ery^r^	[27]
pEF25-*mdxR*	Complementation plasmid for Δ*mdxR*; pEF25 carrying gene *mdxR*	This study

### Construction of deletion mutants and *in trans* complementation

Markerless gene deletion mutants in the *mdxR* gene (locustag: EfmE1162_2133) and the *mdxABCD-pulA* gene cluster (locustag: EfmE1162_0366 - EfmE1162_0370) were created via the Cre-*lox* recombination system as previously described [25,26]. Briefly, the 5′ and 3′ flanking regions (approximately 500 bp each) of the target genes were PCR amplified with the primers in Table 2. The two flanking regions were then fused together by fusion PCR (generating an EcoRI site between both fragments) and cloned into pWS3 [9], resulting in pDEL1a and pDEL2a. Then a gentamicin-resistance cassette which was flanked by *lox66*- and *lox71*-sites [26] was cloned into the EcoRI site that was generated between the 5′ and 3′ flanking regions in pDEL1a and pDEL2a, respectively. The resulting plasmids pDEL1b and pDEL2b were then electrotransformed into *E. faecium* E1162. Marked mutants were obtained by growing the gentamicin-resistant transformants at appropriate temperatures supplemented with appropriate antibiotics [26]. The plasmid pWS3-Cre [26], carrying a gene encoding Cre recombinase, was introduced into the marked mutant by electroporation and further culturing for the removal of the gentamicin resistance cassette and subsequent loss of pWS-Cre was performed as previously described [26]. Excision of the gentamicin resistance cassette and loss of pWS3-Cre was verified by PCR using primers listed in Table 2.

**Table 2:** Primers used in this study.

**Primer**	**Sequence** ^a^
delete_XhoI_mdxR_up_F	5′-CCGC T C G A Gcctgcacctttggaatatgg-3′
delete_EcoRI_mdxR_up_R	5′-AACCTTGACTCGCCCCTTG A A T T Cacgttttgcaacatctgct-3′
delete_EcoRI_mdxR_dn_F	5′-G A A T T Caaggggcgagtcaaggttat-3′
delete_XmaI_mdxR_dn_R	5′-CCCC C C G G Gtgattggtaatggccggtat-3′
check_mdxR_up	5′-catgatcagcttgcagttgg-3′
check_mdxR_dn	5′-gtgtcaacagatgcgtttcg-3′
delete_XhoI_mdxABCD-pulA_up_F	5′-CCGC T C G A Gtgcttgctgataagcatcgt-3′
delete_EcoRI_mdxABCD-pulA_up_R	5′-cgaccggaaagtgaaG A A T T Caataggttccatggcagcag-3′
delete_EcoRI_mdxABCD-pulA_dn_F	5′-ggaacctattG A A T T Cttcactttccggtcgatgat-3′
delete_XmaI_mdxABCD-pulA_dn_R	5′-CCCC C C G G Gttcctagaccgctgacacct-3′
check_mdxABCD-pulA_up	5′-tctttctttggcagccattt-3′
check_mdxABCD-pulA_dn	5′-gacagacaacaaccgatctgaa-3′
qPCR_mdxR_F	5′-agccgacagcaacagtctga-3′
qPCR_mdxR_R	5′-gctcgccgttcaagcattat-3′
qPCR_malP_F	5′-agcgcagcaagcagaaaaag-3′
qPCR_malP_R	5′-cttccatcgttctgcccaag-3′
qPCR_malT_F	5′-gaatcggtgcgctttcttgt-3′
qPCR_malT_R	5′-cgtggcattgattcttgctg-3′
qPCR_tuf_F	5′-TACACGCCACTACGCTCAC-3′
qPCR_tuf_R	5′-AGCTCCGTCCATTTGAGCAG-3′
pAT392_EcoRI_lox66_genta_F	5′-AGGG A A T T CTACCGTTCGTATAGCATACATTATACGAAGTTATG ATAAACCCAGCGAACCATTTGAGG-3′
pAT392_EcoRI_lox71_genta_R	5′-CTCCG A A T T CTACCGTTCGTATAATGTATGCTATACGAAGTTATT CAATCTTTATAAGTCCTTTTATAA-3′

^a^Restriction sites are underlined.

An *in trans* complementated strain (Δ*mdxR+mdxR*) of the *mdxR* deletion mutant (Δ*mdxR*) was generated as previously described [26,27]. The gene *mdxR* was amplified by PCR from the genomic DNA of E1162 using the primers listed in Table 2. The PCR product was cloned into the shuttle vector pEF25 [27]. The resulting plasmid, pEF25-*mdxR*, was introduced into the Δ*mdxR* mutant strain by electroporation as described above [26].

### Determination of growth curves

A BioScreen C instrument (Oy Growth Curves AB, Helsinki, Finland) was used to determine the growth of *E. faecium* strains on starch, maltodextrin, maltose and glucose. Strains were grown overnight in BHI containing appropriate antibiotics at 37° C. Subsequently, cells were inoculated at an initial OD_660_ of 0.0025 in M1 medium. M1 is a semi-defined medium in which *E. faecium* is hardly able to grow when no carbon source is added to the medium [9]. Here, M1 medium was supplemented with starch, the maltodextrin maltoheptaose and maltotetraose, maltose or glucose (2.5 g/l) as carbon sources (all carbohydrates were purchased from Sigma-Aldrich). Cultures were incubated in the Bioscreen C system at 37° C with continuous shaking. The absorbance at 600nm (A_600_) was recorded every 15 min for 12 hours. Each experiment was performed in triplicate.

### Transcriptome profiling

Transcriptome comparisons were performed between the parental strain wild-type E1162 and mutant strain Δ*mdxR* in two conditions. Both strains were grown to mid-exponential (OD_660_ = 0.3) phase in BHI and in M1 supplemented with maltose (M1+maltose), and then RNA isolation, cDNA synthesis and hybridization were performed as described in our previous work [26]. Analysis for statistical significance were performed using the Web-based VAMPIRE microarray suite (http://sasquatch.ucsd.edu/vampire/) as described previously [28,29]. A gene of which all four probes on the microarray were identified as differentially expressed with a false discovery rate <0.05, was classified as significantly differentially expressed between samples. In addition, genes which exhibited an expression between 0.5- and 2-fold different from the wild-type were deemed biologically insignificant and were filtered out. This experiment was performed with four biological replicates.

The microarray data generated for the transcriptome analysis in this study have been deposited in the ArrayExpress database (http://www.ebi.ac.uk/arrayexpress) under accession numbers E-MEXP-3759 (M1+maltose) and E-MEXP-3760 (BHI).

### Quantitative real-time RT-PCR (qRT-PCR)

The total RNA samples of the transcriptome profiling experiment were also used for qRT-PCR. The absence of genomic DNA was verified by PCR prior to reverse transcription. The cDNA was synthesized from total RNA (~1.0 µg) by using the Superscript III First-Strand Synthesis System (Invitrogen, Breda, The Netherlands) according to the manufacturer’s instructions. Using synthesized cDNAs, qRT-PCR was performed using Maxima SYBR Green/ROX qPCR Master Mix (Fermentas, Sankt Leon-Rot, Germany) and a StepOnePlus instrument (Applied Biosystems, Nieuwekerk a/d IJssel, The Netherlands) with the following program: 95° C for 10 min, and subsequently 40 cycles of 95° C for 15 sec, 55° C for 1 min. The expression of *tufA* was used as a housekeeping control [28]. The Ct value of each sample was normalized by the amplification efficiency. The relative transcript level (fold difference relative to *tufA*) of the tested genes were calculated by using the normalized Ct value of the *tufA* housekeeping control minus the normalized Ct value of the tested genes. The resulting value represents a log_2_ transformed fold difference in gene expression. Statistical significance between wild-type and mutant was assessed by the unpaired two-tailed Student’s *t*-test. This experiment was performed with four biological replicates.

## Results

### Identification of genes putatively involved in maltodextrin utilization in *E. faecium*

**Figure 1:**
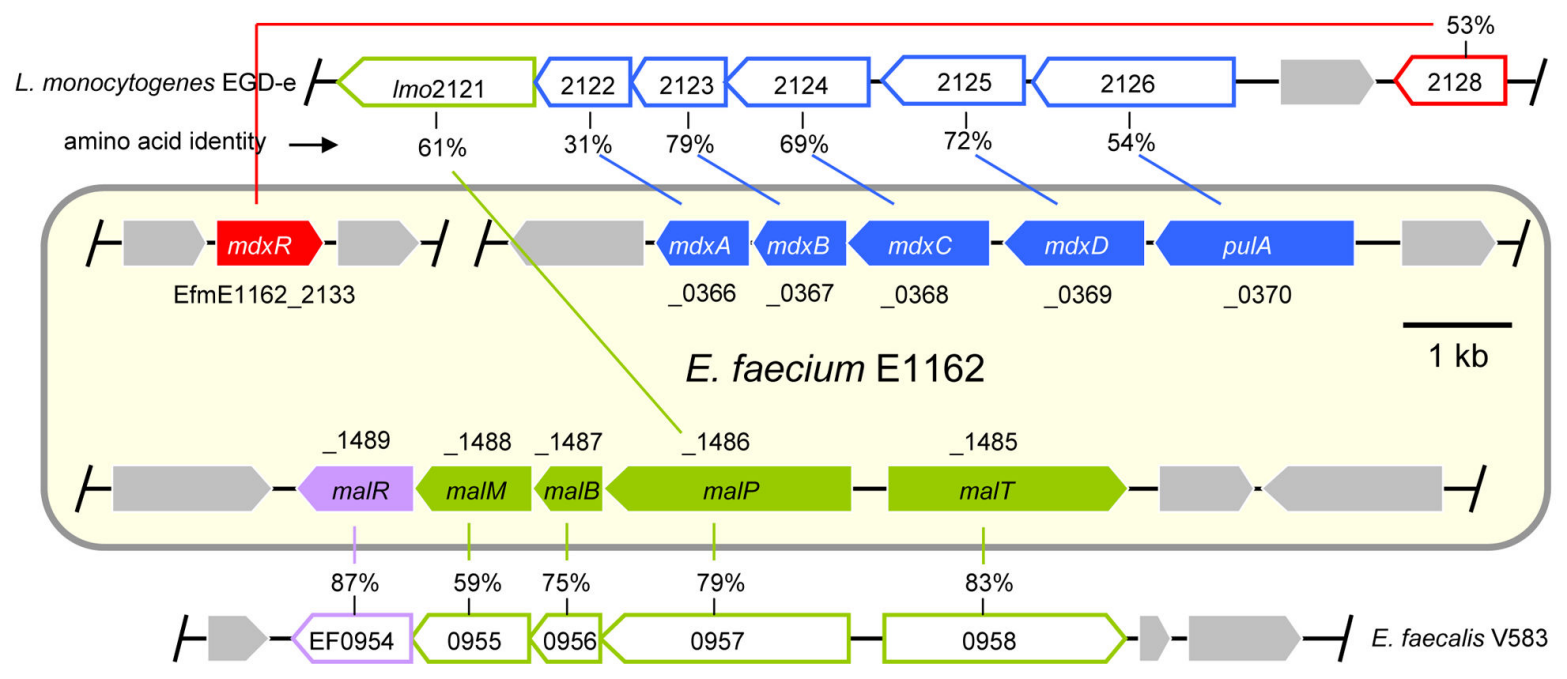
Schematic representation of the gene clusters involved in maltodextrin and maltose utilization of *E. faecium* E1162. Genes are represented by arrows (drawn to scale). Genes putatively encoding proteins involved in maltodextrin transport and/or metabolism are indicated in blue or in red. Genes predicted to be involved in the uptake and/or metabolism of maltose are indicated in green or in purple. The genes that encode putative transcriptional regulators are indicated in red or purple. The grey arrows represent the adjacent genes that are not predicted to be involved in maltodextrin or maltose utilization. Gene names, without the EfmE1162-prefix (omitted for reasons of space), are indicated in the arrows and the gene locus tags are indicated above or below the arrows. The homologs in *L. monocytogenes* EGD-e or in *E. faecalis* V583 are shown with corresponding colors above or below the gene clusters of *E. faecium*. The gene locus tags of the homologs are indicated in the arrows. Lines link the homologous genes with corresponding genes in *E. faecium* and amino acid identities are indicated.

The genome sequence of *E. faecium* strain E1162 was analyzed to identify genes potentially responsible for the utilization of maltodextrin and maltose in *E. faecium*. A search of the E1162 genome for the orthologs of the *L. monocytogenes* and *B. subtilis* maltodextrin utilization proteins led to the identification of a gene cluster (locus tags EfmE1162_0366 - EfmE1162_0370; here termed *mdxABCD*-*pulA*), that is predicted to encode maltose/maltodextrin ABC transporter proteins and a neopullulanase which is predicted to hydrolyze the α-1,4 linkages in starch. The encoded proteins are homologous to the maltodextrin utilization proteins of *L. monocytogenes* EGD-e (amino acid identity: 31%-79%) [20] and *B. subtilis* 168 (amino acid identity: 25%-72%) [18] (Figure 1). In *L. monocytogenes* EGD-e, a regulator gene *lmo2128* is located immediately upstream of the maltodextrin utilization gene cluster. In *E. faecium* E1162, a gene (locus tag: EfmE1162_2133, here termed *mdxR*) encoding a LacI family transcriptional regulator shares the highest amino acid identity (53%) with Lmo2128. The *mdxR* gene is located on a different contig and consequently is not in the immediate vicinity of the *mdxABCD*-*pulA* gene cluster. Analysis of the complete genome sequence of *E. faecium* Aus0004 [30] showed that *mdxR* and the *mdxABCD*-*pulA* gene cluster are located at a distance of 28 kb. The last gene (*lmo2121*) in the maltodextrin utilization gene cluster of *L. monocytogenes* EGD-e encodes a maltose phosphorylase, however the gene (EfmE1162_1486, termed *malP* here) that is homologous to *lmo2121* is not part of the *mdxABCD*-*pulA* gene cluster in E1162. In *E. faecium* Aus0004, *malP* and the *mdxABCD*-*pulA* gene cluster are spaced 48 kb apart. Evidently, there are major differences in genomic organization between the maltodextrin utilization gene clusters of *E. faecium* and *L. monocytogenes*, possibly reflecting functional differences in the metabolism of maltodextrins and maltose between the two organisms.

BLAST analysis showed that all genes of the *mdxABCD*-*pulA* gene cluster are conserved (with amino acid identities >82%) in 66 of the 68 *E. faecium* genomes available (on 16 October 2012) at NCBI Genomes, but no homologous gene cluster is present in *E. faecalis*. The observation that *E. faecalis* strains without this gene cluster could also grow in M1 with maltodextrin (data not shown) indicates that *E. faecalis* has different maltodextrin utilization mechanisms than *E. faecium*. The functional conservation of maltodextrin utilization in *E. faecium* and *E. faecalis* indicates that these traits have been conserved throughout the evolution of these organisms and thus likely contribute to fitness of these commensal bacteria.

### Deletion of *mdxR* and the *mdxABCD-pulA* gene cluster impair growth on maltodextrin

**Figure 2:**
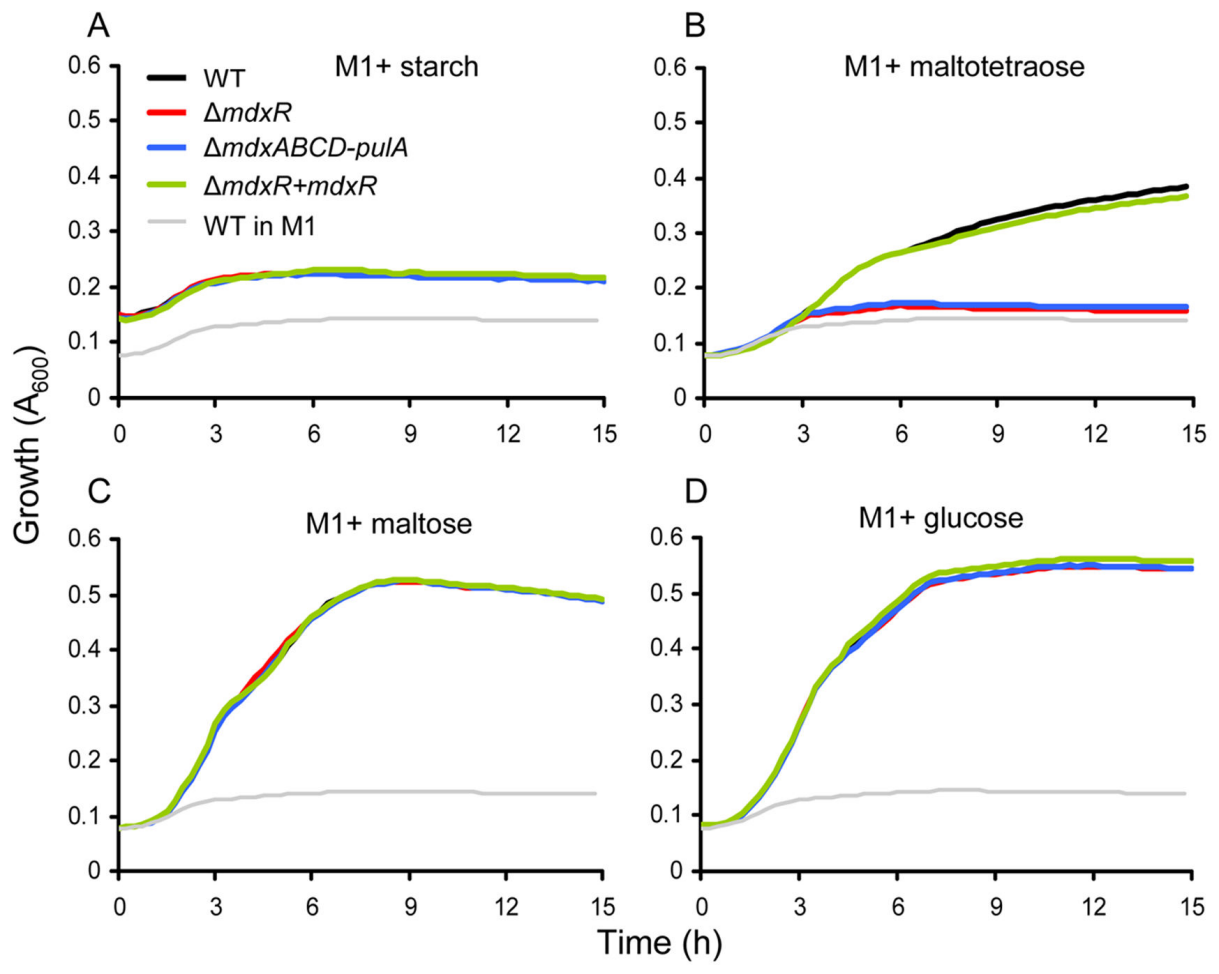
Growth of *E. faecium* on starch, maltotetraose, maltose and glucose. Growth curves of *E. faecium* E1162 wild-type (black), its isogenic mutants Δ*mdxR* (red) and Δ*mdxABCD-pulA* (blue), and the *in trans* complemented strain Δ*mdxR+mdxR* (green) on starch (panel A), maltotetraose (panel B), maltose (panel C) and glucose (panel D) are shown. The growth curve of E1162 wild-type in M1 was shown in grey as a negative control. Overnight cultures were inoculated at an initial OD_660_ of 0.0025 into 300 µl semi-defined minimal medium M1 [9], M1 supplemented with 2.5 g/l of starch, maltotetraoise, maltose or glucose as sole carbon source, respectively, and then incubated in the Bioscreen C system at 37° C with continuous shaking. The absorbance of 600nm (A_600_) was recorded every 15 min for 12 hours. Growth curves are mean data of three independent experiments. Note that A_600_ at the start of the experiment is higher in M1 + starch than in the other conditions, due to increased turbidity of the medium containing starch.

To determine the role of the *mdxABCD-pulA* gene cluster in the ability to grow on maltodextrin, a markerless deletion mutant (Δ*mdxABCD-pulA*) of all five genes in the *mdxABCD*-*pulA* gene cluster was constructed in *E. faecium* E1162. Additionally, a deletion mutant of the gene (*mdxR*) putatively encoding a transcriptional regulator involved in regulating expression of the *mdxABCD*-*pulA* gene cluster was also generated to characterize its role in gene regulation and carbohydrate utilization in *E. faecium* E1162. The mutant Δ*mdxR* was complemented *in trans* (Δ*mdxR+mdxR*), but the *in trans* complemented strain for mutant Δ*mdxABCD-pulA* could not be constructed, presumably due to the large size (6.3 kb) of the DNA fragment encompassing this gene cluster. Growth of *E. faecium* E1162 wild-type (WT), the isogenic mutants and the complemented strain on M1 [9] supplemented with starch, maltodextrin (in the form of maltotetraose), maltose or glucose were determined (Figure 2). The strains did not exhibit appreciable growth on M1 with starch, demonstrating that E1162 could not directly utilize starch. All strains showed identical growth in M1 with glucose, indicating that the introduced mutations did not cause a general growth defect. However, in M1 supplemented with maltotetraose, the growth of the Δ*mdxR* and Δ*mdxABCD–pulA* mutants were impaired, while in M1 supplemented with maltose the growth of these two mutants was comparable to wild-type and the complemented strain. The growth of the Δ*mdxR* and Δ*mdxABCD–pulA* mutants was also impaired in M1 supplemented with maltoheptaose and other maltodextrins with dextrose equivalents of 4.0-7.0, 13-17 and 16.5-19.5 (data not shown). These results show that the *mdxABCD*-*pulA* gene cluster of *E. faecium* is essential for the metabolism of maltodextrin but not for maltose.

### MdxR positively regulates the gene expression of the *mdxABCD-pulA* gene cluster

In *L. monocytogenes* and *B. subtilis*, the expression of the maltodextrin/maltose utilization systems is induced by maltose or maltodextrin [18,20]. To identify the genes that are controlled by MdxR in *E. faecium*, the transcriptome of the Δ*mdxR* mutant was first compared to the transcriptome of its parental strain *E. faecium* E1162 grown to mid-exponential phase in M1 supplemented with maltose (Table 3). We observed eight genes that were significantly lower expressed and four genes that were significantly higher expressed in the Δ*mdxR* mutant strain in comparison to the parental strain during growth in M1 with maltose. Almost all of these genes have a putative role in carbohydrate metabolism, indicating that MdxR does not act globally but rather is specific for a relatively small number of genes. All the genes of the *mdxABCD*-*pulA* gene cluster were expressed at lower levels in the Δ*mdxR* mutant, which confirmed the prediction that MdxR regulates the transcription of this cluster of genes. Three other genes (EfmE1162_1270, EfmE1162_1401, and EfmE1162_1402, which are not located in the immediate vicinity of the *mdxABCD*-*pulA* gene cluster were also expressed at lower levels in the *mdxR* deletion mutant. EfmE1162_1270 and EfmE1162_1401 were annotated as encoding oligo-1,6-glucosidases that share 43% and 51% amino acid identity with the MalL protein of *B. subtilis* 168. MalL is involved in the breakdown of maltodextrin in *B. subtilis* 168 [18] which suggests that EfmE1162_1270 and EfmE1162_1401 also have a role in the metabolism of maltodextrins in *E. faecium*. EfmE1162_1402 is predicted to encode a β-glucosidase that hydrolyzes the β-1,4 bonds of sugars like cellobiose, thereby releasing glucose. There were four genes expressed at higher levels in Δ*mdxR* than in wild-type E1162 during growth in M1+maltose. Three of these genes (EfmE1162_1412 - EfmE1162_1414) were predicted to be involved in glycerol utilization. However, both wild-type E1162 and Δ*mdxR* were unable to grow in M1 supplemented with glycerol in aerobic or anaerobic conditions (data not shown), which was consistent with the previously reported inability of *E. faecium* to grow on glycerol as carbon source [31]. Possibly, *E. faecium* may be able to grow on glycerol in conditions that were not tested in this or previous studies and the functions of EfmE1162_1412 - EfmE1162_1414 thus remain to be determined.

**Table 3:** Comparative transcriptome analysis of *E. faecium* E1162 and the Δ*mdxR* mutant during mid-exponential growth in M1+maltose and BHI.

**LocusTag** ^a^	**Accession code**	**Gene name**	**Annotation**	**Expression ratio Δ*mdxR*/WT**	
				**M1+maltose**	**BHI**
EfmE1162_0366	ZP_06676211	*mdxA*	conserved hypothetical protein	0.16	-^*b*^
EfmE1162_0367	ZP_06676212	*mdxB*	maltose/maltodextrin ABC transporter, permease protein	0.12	-
EfmE1162_0368	ZP_06676213	*mdxC*	maltose/maltodextrin ABC transporter, permease protein	0.27	-
EfmE1162_0369	ZP_06676214	*mdxD*	maltose/maltodextrin ABC transporter, binding protein	0.27	-
EfmE1162_0370	ZP_06676215	*pulA*	Neopullulanase	0.34	-
EfmE1162_1270	ZP_06677115		oligo-1,6-glucosidase	0.23	-
EfmE1162_1401	ZP_06677246		oligo-1,6-glucosidase	0.13	-
EfmE1162_1402	ZP_06677247		beta-glucosidase	0.16	-
EfmE1162_0373	ZP_06676218		conserved hypothetical protein	2.40	-
EfmE1162_1412	ZP_06677257		glycerol kinase	3.05	-
EfmE1162_1413	ZP_06677258		aerobic glycerol-3-phosphate dehydrogenase	3.56	-
EfmE1162_1414	ZP_06677259		glycerol uptake facilitator protein	3.94	-
EfmE1162_0318	ZP_06676163		hypothetical protein	-	0.46
EfmE1162_0408	ZP_06676253		hypothetical protein	-	0.46
EfmE1162_0932	ZP_06676777		hypothetical protein	-	0.40

^a^Genes (excluding *mdxR*) exhibiting significantly different (False Discovery Rate <0.05 and fold-change in expression <0.5 or >2) expression between E1162 wild-type and Δ*mdxR* during mid-exponential growth (OD_660_ = 0.3) in M1+maltose or in BHI are included. This experiment was performed with four biological replicates.

^b^No significant difference in gene expression.

As a control, we also performed a transcriptome comparison analysis between the wild-type E1162 and the Δ*mdxR* mutant strain in BHI (which we routinely use to culture *E. faecium* and contains 2 g/l glucose). Only three genes encoding hypothetical proteins were expressed at slightly lower levels in Δ*mdxR* than in wild-type E1162, indicating that regulation by MdxR is unimportant in a rich, glucose-containing medium.

### The transcription of a putative maltose utilization gene cluster was induced in M1+maltose and expressed independently of *mdxR*

Our results above showed that inactivation of the *mdxABCD*-*pulA* gene cluster of E1162 impaired the growth on maltodextrin but had no effect on the growth on maltose, suggesting that E1162 possesses a maltose utilization system which works independently of the maltodextrin utilization system. In *E. faecalis* a gene cluster composed of five genes has been identified as being responsible for maltose uptake and utilization [32,33]. A homology search in E1162 for the orthologs of this gene cluster identified a gene cluster (EfmE1162_1485 - EfmE1162_1489; here named *malRMBPT*) which is predicted to encode five proteins with amino acid identities ranging from 59% to 87% to their homologs in *E. faecalis* V583 (Figure 1), and this gene cluster is conserved among all the available genomes *of E. faecium* (amino acid identities >96%).

**Figure 3:**
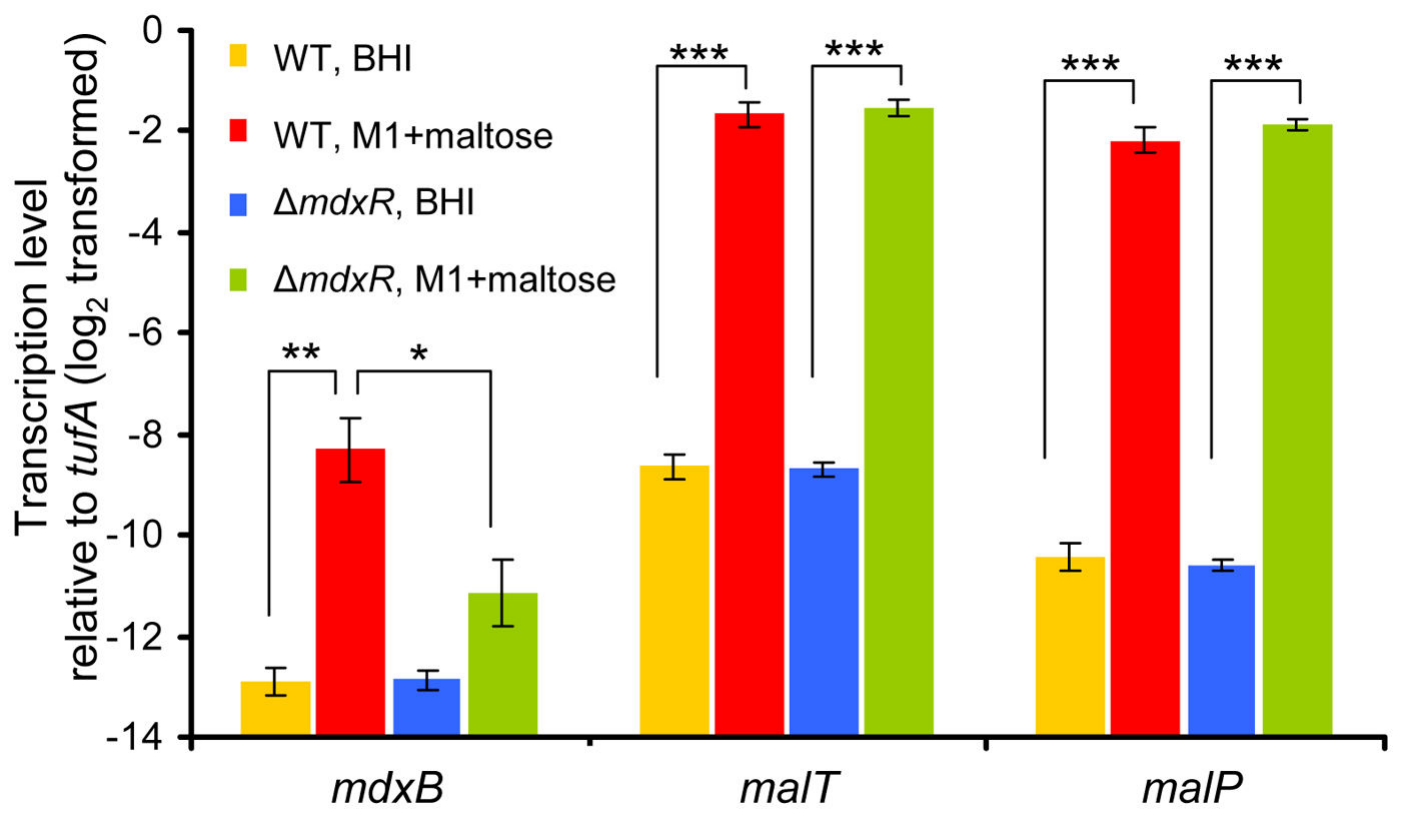
qRT-PCR analysis of *mdxB, malT* and *malP* expression ratios in wild-type E1162 and its isogenic mutant Δ*mdxR* grown in BHI and maltose. The data from the qRT-PCR were normalized using *tufA* as an internal standard [28]. The differences in gene expression (log_2_-transformed data) relative to *tufA* are shown. Error bars represent the standard deviation of the mean of four biological replicates. Asterisks represent significant differences (*: *P*<0.01, **: *P*<0.001, ***: *P*<0.0001, as determined by an unpaired two-tailed Student’s *t*-test) between the indicated samples.

We used qRT-PCR to analyze the expression of *mdxB* (putatively encoding the permease component of the maltodextrin ABC transporter) and two genes (*malT* and *malP*) of the maltose utilization gene cluster in wild-type E1162 and its isogenic mutant Δ*mdxR* grown in BHI and in M1+maltose (Figure 3). We found that, in both wild-type and mutant, the transcription of *mdxB* was at equally low level when grown in BHI, but was strongly upregulated when grown in M1+maltose. Consistent with the observations in the transcriptome analysis, the transcription of *mdxB* was significantly lower (0.14 fold) in Δ*mdxR* than in wild-type E1162 when grown in M1+maltose. In both E1162 and the *mdxR* mutant, the transcription of *malT* and *malP* was greatly induced in M1+maltose compared to BHI. These qPCR data indicate that the EfmE1162_1485 - EfmE1162_1489 gene cluster is regulated independently of MdxR and may be involved in maltose utilization in *E. faecium*, similar to its homologous gene cluster in *E. faecalis* [32].

## Discussion

Carbohydrate utilization is a fundamental metabolic function in bacteria and is an important determinant for niche-adaptation by commensal bacteria [9,34,35]. In this work, we have identified the genetic determinants of **E. faecium** that contribute to its ability to metabolize maltodextrin which is present as a breakdown product of starch in the digestive tract of humans and animals. Our results confirm previous data by showing that *E. faecium* cannot grow on starch [36], indicating that *E. faecium* can only benefit from the degradation products of starch due to the action of amylolytic enzymes, which are produced by the host and other gut commensals. We show that the *mdxABCD-pulA* gene cluster of *E. faecium* confers the ability to grow on maltodextrin. The deletion of *mdxABCD-pulA* did not affect the growth rate on maltose, suggesting that *E. faecium* has an independent system for the utilization of maltose. Similarly in *B. subtilis* , maltose is transported by maltose-specific phosphotransferase system, while maltodextrin is transported by a maltodextrin-specific ABC transporter [18]. In contrast, *L. monocytogenes* takes up both maltose and maltodextrin by the same ABC transporter [20]. Therefore, we propose that, similar to the situation in *B. subtilis* , the proteins encoded by the *mdxABCD-pulA* gene cluster are exclusively involved in maltodextrin transport and metabolism, and do not have a role in maltose metabolism.

Our transcriptome analysis and qRT-PCR experiments showed that the expression of the *mdxABCD-pulA* gene cluster and the putative maltose utilization gene cluster *malRMBPT* is induced by maltose and repressed in medium which contains a rapidly-metabolized sugar such as glucose. The expression level of the putative maltose utilization gene cluster is considerably higher in M1+maltose than in BHI, but is unaffected by the deletion of *mdxR*. The *mdxB* gene of the maltodextrin transport and metabolism gene cluster was expressed at significantly higher levels in wild-type E1162 than in Δ*mdxR* when grown in M1+maltose, but, interestingly, in Δ*mdxR*expression of *mdxB* is still higher in M1+maltose than in BHI, suggesting that MdxR is not the sole regulator governing expression of the *mdxABCD*-*pulA* gene cluster. A possible explanation is that transcription of the *mdxABCD*-*pulA* gene cluster is coregulated by MdxR and HPr kinase/P-Ser-HPr phosphatase (HPrK/P) systems, which in *L. monocytogenes* [20] and *L. lactis* [37] represses the expression of genes involved in maltodextrin or maltose utilization in the presence of glucose. Consequently, upregulation of *mdxB* in M1+maltose may partially result from the release of glucose-mediated repression rather than stimulation by maltose. However, this upregulation is insufficient to support the effective growth on maltodextrin, for which MdxR is absolutely required.

*E. faecium* possesses a wide range of carbohydrate metabolic pathways [23] allowing utilization of a variety of sugars. In this study, we have identified an *E. faecium* gene cluster that is responsible for utilization of maltodextrin, a potential important carbon source for *E. faecium* in the gut. These data could contribute to the mechanistic understanding of the lifestyle of *E. faecium* as a gut commensal.
